# Checklist of the Ichneumonidae of Germany (Insecta, Hymenoptera)

**DOI:** 10.3897/BDJ.9.e64267

**Published:** 2021-05-26

**Authors:** Matthias Riedel, Andrei E. Humala, Martin Schwarz, Heinz Schnee, Stefan Schmidt

**Affiliations:** 1 c/o SNSB-Zoologische Staatssammlung München, Bad Fallingbostel, Germany c/o SNSB-Zoologische Staatssammlung München Bad Fallingbostel Germany; 2 Forest Research Institute KRC RAS, Petrozavodsk, Russia Forest Research Institute KRC RAS Petrozavodsk Russia; 3 Biologiezentrum Linz, Linz, Austria Biologiezentrum Linz Linz Austria; 4 c/o SNSB-Zoologische Staatssammlung München, Markkleeberg, Germany c/o SNSB-Zoologische Staatssammlung München Markkleeberg Germany; 5 SNSB-Zoologische Staatssammlung München, München, Germany SNSB-Zoologische Staatssammlung München München Germany

**Keywords:** Ichneumonoidea, distribution, parasitoids, checklist, new records

## Abstract

**Background:**

A revised checklist of the Ichneumonidae of Germany is provided. The list represents an updated version of an earlier checklist published in 2001. The present list includes several records of species that are new for the German fauna and species that were discovered since the last checklist was published. The present checklist was compiled as part of the DNA barcoding projects at the Zoologische Staatssammlung München.

**New information:**

The checklist includes 3,644 species of Ichneumonidae from Germany, with 48 species recorded for the first time. Compared to the checklist published 20 years ago, the number of ichneumonid species recorded from Germany has increased by 312 species.

## Introduction

The Ichneumonidae is the largest family of insects in Germany, with over 3,600 species already recorded for the country and an estimated number of 4,000 species in total (Horstmann 2002a). The family Ichneumonidae and its sister family Braconidae, with about 1,500 German species (Belokobylskij et al. 2003), account for about half of the German species of the insect order Hymenoptera (Völkl and Blick 2004).

The first comprehensive and validated checklist of ichneumonid species that were recorded from Germany was provided by Horstmann (2001a). When compiling the checklist, he included only species that were either described as new after 1945 or, if the species was described before 1945 or if it was mentioned at least once in the entomological literature after WWII. Horstmann (2001a) excluded species that were mentioned only once in the literature prior to 1945, i.e. in the original citation, because, according to him, virtually all of those species represent synonyms. If the species were cited after 1945, he included it only if it was accompanied by a taxonomic treatment. Following this rationale, it was possible for him to compile a checklist that comprises only reliable records while reducing the risk of including species with doubtful status.

The list was compiled as part of DNA barcoding projects at the Zoologische Staatssammlung München (ZSM) that aims at assembling DNA barcode libraries for Germany (German Barcode of Life Project, www.bolgermany.de) and Bavaria (Barcoding Fauna Bavarica). A new checklist was deemed necessary because of the relatively large number of species that were added since the last checklist was published 20 years ago by Klaus Horstmann (2001a) and because of several nomenclatorial changes (e.g. synonymy) and recent modifications to the classification within the family (Broad and Shaw 2005, Bennett et al. 2019, Klopfstein et al. 2019, Santos 2017). Most records of species new to Germany, a few of which are depicted in Fig. 1, resulted from extensive collecting efforts as part of past and ongoing projects on DNA barcoding (Hausmann et al. 2020, Morinière et al. 2016).

## Materials and methods

The present checklist is an updated version of the checklist of German Ichneumonidae provided by Horstmann (2001a). Most additions to the new checklist are based on records that were published since the previous checklist (Horstmann 2001a). Identifications of species that are recorded as new for the German fauna were identified by specialists as part of the ongoing DNA barcoding efforts at the ZSM. Representatives of newly-recorded species are deposited in the ZSM or in the author's private collections.

The classification follows Bennett et al. (2019), with 42 recognised subfamilies and includes recent changes to the subfamily-level classification, in particular, the division of the Cryptinae *sensu lato* into the three subfamilies Ateleutinae, Cryptinae and Phygadeuontinae (Santos 2017) and recognition of the subfamily Neorhacodinae (Bennett et al. 2019). Furthermore, following Klopfstein et al. (2019), the Theroniini is recognised as a tribe of the subfamily Pimplinae, *Pseudorhyssa* is listed under Pimplinae and *Hemiphanes* under Cryptinae.

## Data resources

The present checklist comprises 3,644 species of Ichneumonidae for Germany (Suppl. material 1), including 379 species that were added from the published literature since the checklist by Klaus Horstmann (Horstmann 2001a) and 48 species that are here reported from Germany for the first time (Table 1). Several recent studies, examining phylogenetic relationships amongst subfamilies within the Ichneumonidae, led to changes to the internal classification of the family (e.g. Santos 2017, Bennett et al. 2019, Broad et al. 2018, Klopfstein et al. 2019). Table 2 includes (1) species hereby added to the German fauna, (2) newly-recorded species and (3) species listed under a different name (e.g. synonyms) or species with a different systematic position in Horstmann (2001a).

## Checklists

### Checklist of the Ichneumonidae of Germany

#### Ichneumonidae

Latreille, 1802

5CE9081F-703E-5E54-86F7-9FF5968262D4

##### Notes

For a complete checklist of German Ichneumonidae, see Suppl. material 1.

## Discussion

The first checklist of Ichneumonidae for Germany was presented by Horstmann as part of the "Verzeichnis der Hautflügler Deutschlands" (Dathe et al. 2001). An essential prerequisite for preparing the checklist was the presence of the comprehensive "Taxapad" database that became available in 1999. The database had been continuously updated, with the most recent version released in 2015 (Yu et al. 2016).

Klaus Horstmann initially hesitated with compiling the checklist because of the daunting task and because, in his opinion, a scientifically satisfactory checklist could not be achieved (Horstmann 2002a). The main reason for his scepticism was based on the fact that there was a huge discrepancy between the extremely large number of species and a few specialists working on the family. As an example, he noted that, for the compilation of the checklist of German Sphecidae, 32 specialists were responsible for 247 species, whereas for the 3,332 species of Ichneumonidae, only one specialist was available (Horstmann 2002a). Due to the limited capacity, it was impossible to validate the status of each and every species and, to the present day, many species of Ichneumonidae, in particular, species in genera of the subfamilies Banchinae, Campopleginae, Phygadeuontinae, Ctenopelmatinae and Ichneumoninae, are poorly defined and their identification remains most challenging.

Using the comprehensive keys in Schmiedeknecht (1936) or other keys published in older literature, many of the taxonomically problematic species can somehow be 'identified', but, as Horstmann (2002a) states, it can be assumed with some confidence that many names in the faunistic literature are based on misidentifications and it is estimated that between 10 and 20% of names mentioned in faunistic publications were incorrectly identified (Yu and Horstmann 1997). Horstmann (2002a) noted that the rate of misidentifications is higher with species that usually represent the most interesting parts of a faunistic treatment, i.e. species that are rare and/or difficult to identify.

With these limitations in mind, Horstmann followed a set of rules when preparing the checklist. First and foremost, only original records were considered for compiling the checklist. Faunistic records were excluded if they were suspected to be based on misidentifications, if they were based on varieties of a particular species or if their collecting locality was unclear. For example, species that were recorded from Germany before WWII without giving the exact locality might be extralimital and were, therefore, not included. Species were excluded if the original description was published before 1945 and the species never treated in a taxonomic revision or mentioned in the taxonomic literature after that year. Finally, species were excluded if their records were based on males from genera where males are virtually impossible to identify or genera where males had not been associated with conspecific females. Only in a few cases were these species included on a case by case basis (Horstmann 2002a, Dathe et al. 2001). Species were included if they are still regarded as valid names and the type locality is in Germany.

The present checklist basically follows the same rationale. Species that were added to the current checklist are based on records of reliably identified specimens in the taxonomic and faunistic literature. In a few cases, species not included in Horstmann's checklist were added, based on a recent critical evaluation of the species or the literature source.

The catalogue of Ichneumonidae (Yu and Horstmann 1997, Yu et al. 2016) lists 4,121 species for Germany, a figure that is considerably larger than the number of species in the current checklist. This is partly due to the fact that the catalogue also includes fossils. At the time when Horstmann prepared his checklist, the catalogue listed 3,898 species from Germany, of which 741 were excluded due to the aforementioned reasons (Horstmann 2002a). It is estimated that the presence of about 10% of the species in Germany requires confirmation, partly due to undetected synonymies and, to a much larger extent, because of misidentifications (Horstmann 2002a). Although the status of a number of species could be confirmed for Germany since the last checklist was published, the percentage of species requiring confirmation is probably not much different from the figure given by Horstmann (2002a). On the other hand, even the updated checklist does not include all species occurring in Germany and it is estimated that about half of them are species that are known, but have not been recorded in Germany and the other half represents species that have not yet been formally described.

Townes (1969) estimated that, for the Western Palearctic Region, about 30% of the species of Ichneumonidae are still undescribed. For Germany, this percentage is probably lower, but, assuming that 15-20% or even up to 30% of the German species are undescribed, the estimated number of species occurring in Germany ranges from 4,000 to 4,500 species. Most of these are species in genera with large numbers of undescribed species, including *Campoplex* Gravenhorst, *Diadegma* Förster, *Lissonota* Gravenhorst, *Olesicampe* Holmgren, *Phygadeuon* Gravenhorst and *Stenomacrus* Förster (Horstmann 2002a).

Twenty years after the last checklist was published, the present checklist raises the number of Germany species of Ichneumonidae by 312 species or about nine percent. Closing the gap between the currently-known species and a complete inventory of German species will require substantial efforts in terms of collecting, specimen processing and taxonomic research, but the application of integrative taxonomic approaches that combine independent character systems, including morphology, DNA sequence data and host information, hold promise of expediting the discovery of new species of Ichneumonidae in Germany and the clarification of their taxonomy and biology.

## Supplementary Material

E6862676-2000-55F7-9E7D-ED2B91BE36E3 10.3897/BDJ.9.e64267.suppl1 Supplementary material 1 Checklist of German Ichneumonidae Data type Species checklist File: oo_526684.xlsx https://binary.pensoft.net/file/526684 Riedel, M., Humala, A., Schnee, H., Schwarz, M., Schmidt, S.

XML Treatment for Ichneumonidae

## Figures and Tables

**Figure 1a.**
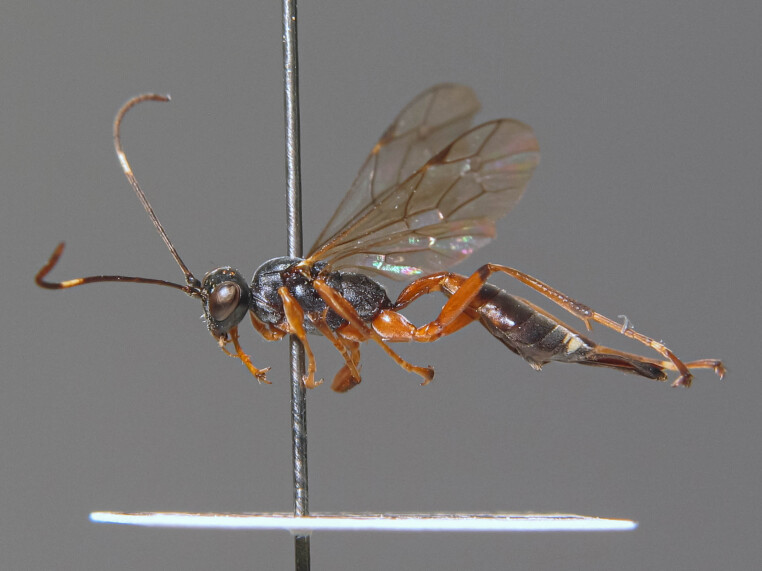
*Thrybius brevispina* (Thms.) (Cryptinae, specimen ID: BC ZSM HYM 09699)

**Figure 1b.**
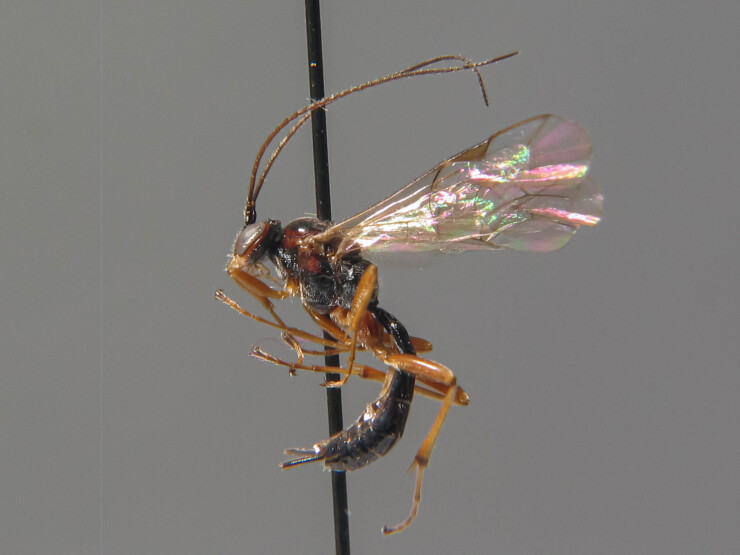
*Mesochorus marginatus* Thms. (Mesochorinae, specimen ID: BC ZSM HYM 19719)

**Figure 1c.**
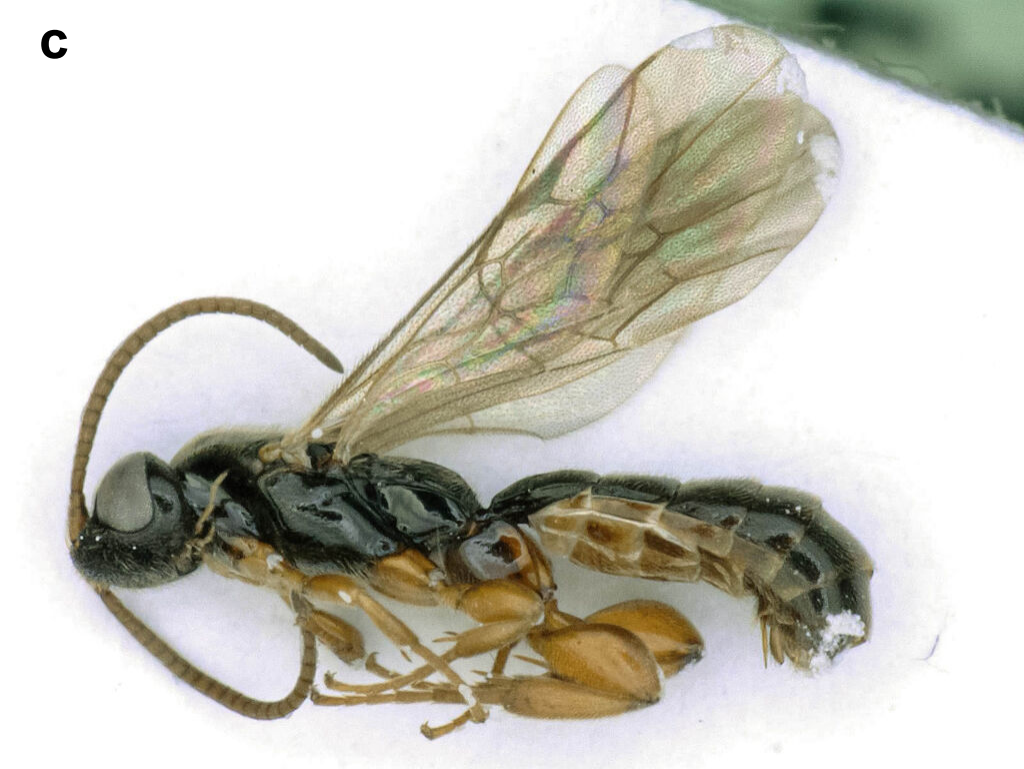
*Hypsicera ecarinata* Tolk. (Metopiinae, specimen ID: BC-ZSM-HYM-24112-G10)

**Figure 1d.**
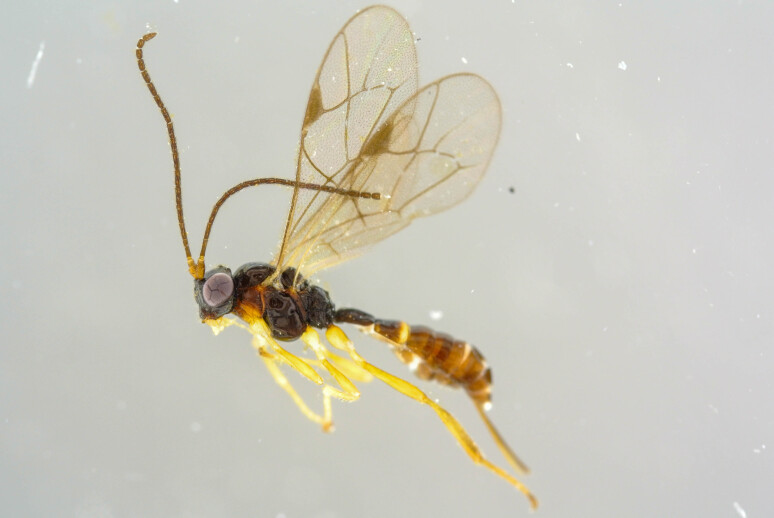
*Aniseres subarcticus* Humala (Orthocentrinae, specimen ID: BC-ZSM-HYM-27568-H10)

**Figure 1e.**
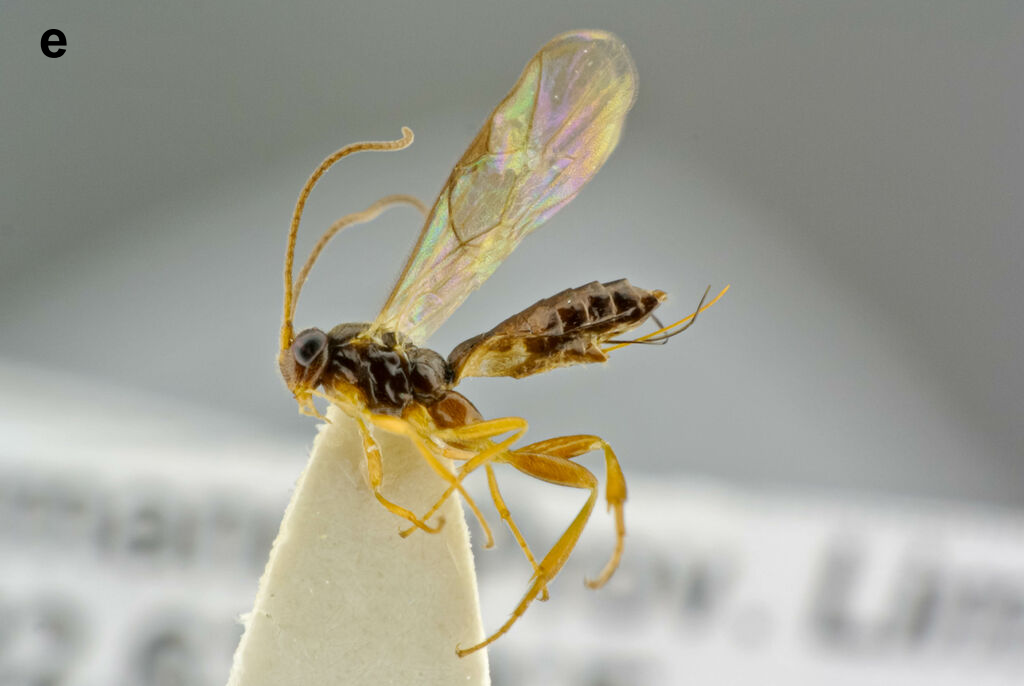
*Batakomacrus subarcticus* Humala (Orthocentrinae, specimen ID: BC-ZSM-HYM-27505-F03)

**Figure 1f.**
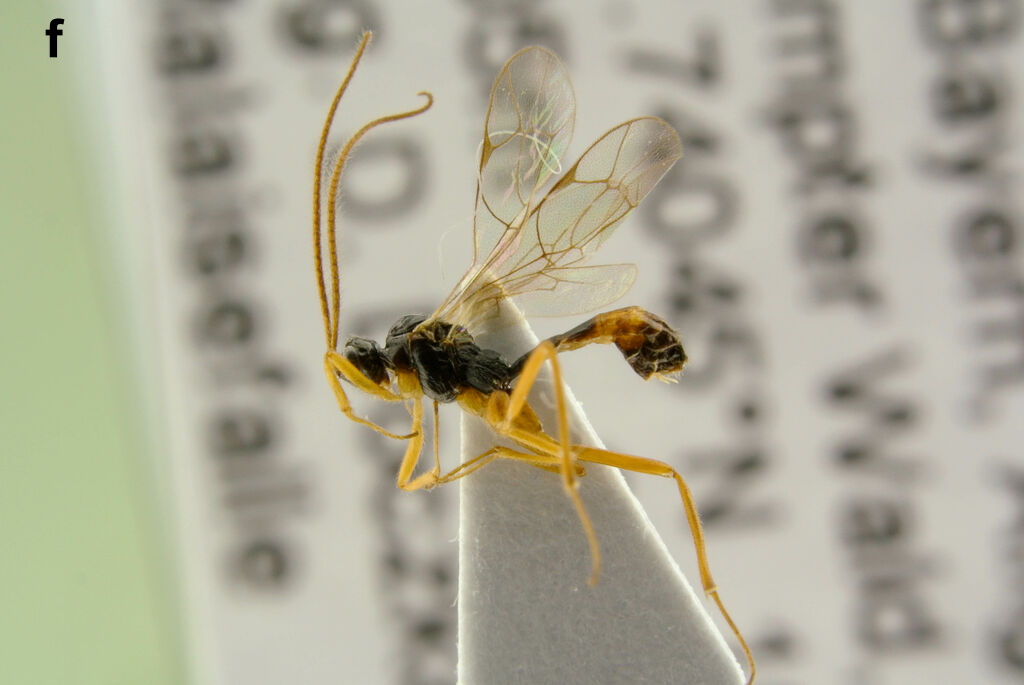
*Megastylus similis* Dasch (Orthocentrinae, specimen ID: BC-ZSM-HYM-22280-A05)

**Table 1.** Number of species of German Ichneumonidae by subfamily in Horstmann (2001a) and in the present checklist, with number of species added from published records and species recorded for the first time from Germany. Number of Cryptinae species in brackets show the number of species in Horstmann (2001a), i.e. Cryptinae *sensu lato.* The number of species in the present list is lower than in the old checklist plus additions and new records if species were deleted or disappeared into synonymy. ^1^in Horstmann (2001a) under Cryptinae, ^2^in Horstmann (2001a) in Paxylommatinae, now placed in Hybrizontinae, ^3^in Horstmann (2001a) placed in Cryptinae and including *Helcostizus restaurator*, that is now placed in Cryptinae, ^4^Phrudinae now placed in Tersilochinae.

**Subfamily**	**Horstmann (2001a)**	**Present checklist**	**Additions**	**New records**
Acaenitinae	15	14		
Adelognathinae	16	23	6	1
Agriotypinae	1	1		
Anomaloninae	57	62	6	1
Ateleutinae	1^1^	1		
Banchinae	178	184	9	
Brachycyrtinae	1	1		
Campopleginae	454	498	55	2
Collyriinae	1	2	2	
Cremastinae	25	26		1
Cryptinae	215	230	17	7
Ctenopelmatinae	386	421	54	1
Cylloceriinae	4	7	1	2
Diacritinae	1	1		
Diplazontinae	61	68	9	
Eucerotinae	4	4		
Hybrizontinae	4^2^	5	1	
Ichneumoninae	602	638	58	6
Lycorininae	1	1		
Mesochorinae	163	175	26	2
Metopiinae	90	92	5	1
Microleptinae	4	3		
Neorhacodinae	1	1		
Ophioninae	25	41	9	6
Orthocentrinae	136	151	11	10
Orthopelmatinae	1	2		1
Oxytorinae	2	2		
Phygadeuontinae	404^3^	468	65	5
Pimplinae	134	146	14	
Phrudinae	5	n/a^4^		
Poemeniinae	11	11	2	
Rhyssinae	8	9	1	
Stilbopinae	6	6		
Tersilochinae	98	113^4^	10	
Tryphoninae	190	208	18	2
Xoridinae	28	29	1	
**Total**	**3332**	**3644**	**379**	**48**

**Table 2.** Species with different status or name compared to the previous checklist by Horstmann (2001a). Status codes: (A) published record, (N) new record for Germany, (H) present in Horstmann (2001a), but name or systematic placement different.

	**Species**	**Status**	**Remarks**
** Adelognathinae **			
	*Adelognathus acantholydae* Kasparyan, 1986	A	Riedel (2018a)
	*Adelognathus cubiceps* Roman, 1925	A	Kasparyan and Kopelke (2009)
	*Adelognathus difformis* Holmgren, 1857	A	Heitland and Pschorn-Walcher (2005)
	*Adelognathus facialis* Thomson, 1883	A	Riedel (2007)
	*Adelognathus marginellus* Holmgren, 1857	N	New record (A. Humala det.)
	*Adelognathus obscurus* Kasparyan, 1986	A	Riedel (2018a)
	*Adelognathus tetratinctorius* (Thunberg, 1824)	A	Riedel (2007)
** Anomaloninae **			
	*Agrypon brachycerum* Hellén, 1950	A	Schnee (2008)
	*Agrypon flaveolatum* (Gravenhorst, 1807)	H	Syn: *Agrypon faciale* Szépligeti (Schnee 2018)
	*Agrypon hinzi* Schnee, 2018	A	Schnee (2018)
	*Agrypon minutum* Bridgman & Fitch, 1884	A	Schmidt and Zmudzinski (2009)
	*Aphanistes wadai* Uchida, 1958	A	Schnee (2018)
	*Barylypa helleni* Schnee, 1989	A	Schnee (2018)
	*Barylypa mesozona* (Förster, 1878)	H	In Horstmann (2001a) as *Barylypa longicornis* (Brauns, 1895); see also Schnee (2008)
	*Barylypa propugnator* (Förster, 1855)	H	In Horstmann (2001a) as *Barylypa insidiator* (Förster, 1878); see also Schnee (2008)
	*Erigorgus varicornis* (Thomson, 1894)	A	Schnee (2008)
	*Heteropelma grossator* Shestakov, 1923	N	New record (H. Schnee det.)
** Ateleutinae **			
	*Ateleute linearis* Förster, 1871	H	Raised to subfamily level from tribe Cryptini, subtribe Ateleutina, by Santos (2017)
** Banchinae **			
	*Cryptopimpla alpigena* (Strobl, 1902)	A	Riedel 2018a
	*Cryptopimpla anomala* (Holmgren, 1860)	H	In Horstmann (2001a) as *Lissonota anomala* Holmgren, 1860
	*Cryptopimpla subfumata* (Thomson, 1877)	A	Horstmann (2008a)
	*Lissonota albicoxis* Kriechbaumer, 1888	A	Riedel (2018a)
	*Lissonota distincta* Bridgman, 1889	A	Schmidt and Zmudzinski (2009)
	*Lissonota funebris* Habermehl, 1923	A	Riedel (2018a)
	*Lissonota oculatoria* (Fabricius, 1798)	H	In Horstmann (2001a) as *Lissonota elector* Gravenhorst, 1829
	*Lissonota parasitellae* Horstmann, 2003	A	Horstmann (2003a)
	*Lissonota sahlbergi* Hellén, 1915	A	Riedel 2018a
	*Rynchobanchus flavopictus* Heinrich, 1937	A	Riedel (2002)
	*Diblastomorpha cylindrator* (Fabricius, 1787)	H	In Horstmann (2001a) as *Glypta cylindrator* (Fabricius, 1787)
	*Glypta brevipetiolata* Thomson, 1889	A	Horstmann (2008a)
** Campopleginae **			
	*Bathyplectes clypearis* (Horstmann, 1974)	A	Schmidt et al. (2011)
	*Campoletis cubicator* Aubert, 1974	A	Riedel (2017)
	*Campoletis ensator* (Gravenhorst, 1829)	A	Riedel (2017)
	*Campoletis flagellator* Riedel, 2017	A	Riedel (2017)
	*Campoletis hinzi* Riedel, 2017	A	Riedel (2017)
	*Campoletis latrator* (Gravenhorst, 1829)	A	Horstmann (2008a)
	*Campoletis nigritrochantellus* Riedel, 2017	A	Riedel (2017)
	*Campoletis pectalis* Riedel, 2017	A	Riedel (2017)
	*Campoletis procerus* (Brischke, 1880)	H	In Horstmann (2001a) as *Campoplex procerus* (Brischke, 1880)
	*Campoletis rapax* (Gravenhorst, 1829)	H	In Horstmann (2001a) as *Campoletis erythropus* (Thomson, 1887)
	*Campoletis rubidatae* Riedel, 2017	A	Riedel (2017)
	*Campoletis rufifasciatae* Riedel, 2017	A	Riedel (2017)
	*Campoletis variator* Riedel, 2017	A	Riedel (2017)
	*Campoletis vimmeri* (Gregor, 1938)	A	Riedel (2017)
	*Campoplex anomalus* Gravenhorst, 1829	A	Horstmann (2008a)
	*Campoplex formosanae* Horstmann, 2012	A	Horstmann (2012a)
	*Campoplex nigrifemur* (Szépligeti, 1916)	A	Schmidt et al. (2012)
	*Campoplex psammae* (Morley, 1915)	A	Horstmann (2012a)
	*Campoplex psilopterus* Gravenhorst, 1829	A	Horstmann (2000d)
	*Campoplex restrictor* Aubert, 1960	A	Schmidt et al. (2011)
	*Campoplex rufipes* Gravenhorst, 1829	A	Horstmann (2008b)
	*Campoplex serratellae* Horstmann, 2012	A	Horstmann (2012a)
	*Casinaria dubia* Tschek, 1871	A	Riedel (2018c)
	*Casinaria pallipes* Brischke, 1880	A	Riedel (2018c)
	*Casinaria paramorionella* Riedel, 2018	A	Riedel (2018c)
	*Casinaria pyreneator* Aubert, 1960	A	Riedel (2018c)
	*Casinaria subglabra* Thomson, 1887	A	Riedel (2018c)
	*Casinaria tegulata* Riedel, 2018	A	Riedel (2018c)
	*Casinaria trochanterator* Aubert, 1960	A	Schmidt et al. (2011)
	*Cymodusa ancilla* (Seyrig, 1927)	N	New record (H. Schnee det.)
	*Cymodusa convergator* (Aubert, 1972)	A	Horstmann (2013)
	*Cymodusa tibialis* Dbar, 1985	A	Riedel (2007)
	*Diadegma crassiseta* (Thomson, 1887)	A	Riedel (2018a)
	*Diadegma ericinellae* Horstmann, 2013	A	Horstmann (2013)
	*Diadegma naryciae* Horstmann, 2008	A	Horstmann (2008a)
	*Dolophron nemorati* Horstmann, 1978	A	Schmidt et al. (2011)
	*Dusona bicoloripes* (Ashmead, 1906)	H	In Horstmann (2001a) as *Dusona foersteri* (Roman, 1942)
	*Dusona blanda* (Förster, 1868)	H	In Horstmann (2001a) as *Dusona remota*
	*Dusona carpathica* (Szépligeti, 1916)	H	In Horstmann (2001a) as *Dusona remota* (Förster, 1868)
	*Dusona flagellator* (Fabricius, 1793)	H	In Horstmann (2001a) as *Dusona debilis* (Förster, 1868) and *D. heterocera* (Förster, 1868)
	*Dusona hastulatae* Horstmann, 2009	A	Horstmann (2009a)
	*Dusona mercator* (Fabricius, 1793)	H	In Horstmann (2001a) as *Dusona oxyacanthae* (Boie, 1855)
	*Dusona meridionator* Aubert, 1960	A	Horstmann (2011b)
	*Dusona nebulosa* Horstmann, 2004	A	Horstmann (2004)
	*Dusona pulchripes* (Holmgren, 1872)	A	Horstmann (2011b)
	*Dusona prominula* (Förster, 1868)	H	In Horstmann (2001a) as *Dusona contumax* (Förster, 1868)
	*Dusona rubidatae* Horstmann, 2009	A	Horstmann (2009a)
	*Dusona signator* (Brauns, 1895)	A	Schmidt et al. (2011)
	*Enytus apostatus* (Gravenhorst, 1829)	H	In Horstmann (2001a) as *Enytus apostata* (Gravenhorst, 1829)
	*Enytus appositor* (Aubert, 1970)	A	Schmidt et al. (2011)
	*Enytus rufoapicalis* Horstmann, 2004	A	Horstmann (2004), Humala (2004)
	*Hyposoter boops* (Thomson, 1887)	A	Horstmann (2013)
	*Hyposoter caudator* Horstmann, 2008	N	New record (M. Riedel det.)
	*Hyposoter neglectus* (Holmgren, 1860)	H	In Horstmann (2001a) as *Phobocampe neglecta* (Holmgren, 1860)
	*Hyposoter seniculus* (Gravenhorst, 1829)	A	Horstmann (2008a)
	*Lathrostizus clypeatus* (Brischke, 1880)	H	In Horstmann (2001a) as *Lathrostizus sternocerus* (Thomson, 1887)
	*Lathrostizus forticanda* (Thomson, 1887)	A	Horstmann (2004)
	*Lemophagus errabundus* (Gravenhorst, 1829)	H	In Horstmann (2001a) as *Olesicampe errabunda* (Gravenhorst, 1829)
	*Lemophagus pulcher* (Szépligeti, 1916)	A	Haye and Kenis (2004)
	*Meloboris dimicatellae* Horstmann, 2004	A	Riedel (2018a)
	*Meloboris proxima* (Perkins, 1942)	A	Horstmann 2013)
	*Nemeritis elegans* (Szépligeti, 1901)	A	Riedel (2018a)
	*Olesicampe transiens* (Ratzeburg, 1848)	H	In Horstmann (2001a) as *Olesicampe incrassator* (Holmgren, 1856)
	*Olesicampe vetula* (Holmgren, 1860)	A	Horstmann (2008a)
	*Phobocampe brumatae* Horstmann, 2009	A	Schmidt et al. (2011)
	*Phobocampe croceipes* (Marshall, 1876)	A	Sedivy (2004), Horstmann (2007)
	*Phobocampe horstmanni* Sedivy, 2004	A	Sedivy (2004)
	*Phobocampe nigra* Sedivy, 2004	A	Sedivy (2004)
	*Phobocampe quercus* Horstmann, 2008	A	Horstmann (2008a)
	*Porizon albistriae* (Horstmann, 1987)	H	In Horstmann (2001a) as *Phaedroctonus albistriae* (Horstmann, 1987)
	*Porizon humuli* (Horstmann, 1987)	H	In Horstmann (2001a) as *Phaedroctonus humuli* (Horstmann, 1987)
	*Porizon moderator* (Linnaeus, 1758)	H	In Horstmann (2001a) as *Phaedroctonus moderator* (Linnaeus, 1758)
	*Porizon transfuga* (Gravenhorst, 1829)	H	In Horstmann (2001a) as *Phaedroctonus transfuga* (Gravenhorst, 1829)
	*Synetaeris brevicauda* (Horstmann, 1987)	H	In Horstmann (2001a) as *Pyracmon brevicauda* (Horstmann, 1987)
	*Tranosema variabile* Horstmann, 2008	A	Horstmann (2008a)
** Collyriinae **			
	*Collyria trichophthalma* (Thomson, 1877)	A	Schmidt and Zmudzinski (2003)
** Cremastinae **			
	*Temelucha ophthalmica* (Holmgren, 1866)	N	New record (J. Müller det.)
** Cryptinae **			
	*Aptesis perversa* (Kriechbaumer, 1893)	H	In Horstmann (2001a) as *Pleolophus perversus* (Kriechbaumer, 1893)
	*Cratocryptus subpetiolatus* (Gravenhorst, 1829)	A	Riedel et al. (2013)
	*Cubocephalus annulitarsis* (Thomson, 1873)	N	New record (M. Schwarz det.)
	*Cubocephalus fortipes* (Gravenhorst, 1829)	A	Schmidt and Zmudzinski (2003)
	*Cubocephalus lacteator* (Gravenhorst, 1829)	A	Horstmann (2008a)
	*Cubocephalus leucopygus* (Kriechbaumer, 1891)	A	Sawoniewicz (2003)
	*Cubocephalus montanus* (Gravenhorst, 1829)	H	In Horstmann (2001a) as *Aptesis hanseatica* (Habermehl)
	*Cubocephalus sperator* (Müller, 1776)	H	In Horstmann (2001a) as *Pleolophus sperator* (Müller, 1873)
	*Hemiphanes erratum* Humala, 2007	A	Quicke et al. (2009), formerly placed in Orthocentrinae (Klopfstein et al. 2019)
	*Hemiphanes flavipes* Förster, 1871	H	Formerly placed in Orthocentrinae (Klopfstein et al. 2019)
	*Hemiphanes gravator* Förster, 1871	H	Formerly placed in Orthocentrinae (Klopfstein et al. 2019)
	*Oresbius subguttatus* (Gravenhorst, 1829)	H	In Horstmann (2001a) as *Aptesis subguttata* (Gravenhorst, 1829)
	*Schenkia exigua* (Habermehl, 1909)	A	Schmidt and Zmudzinski (2007)
	*Agrothereutes australis* (Habermehl, 1926)	N	New record (M. Schwarz det.)
	*Agrothereutes tibialis* (Thomson, 1873)	N	New record (M. Schwarz det.)
	*Aritranis explorator* (Tschek, 1871)	A	Schmidt and Zmudzinski (2003)
	*Aritranis occisor* (Gravenhorst, 1829)	H	In Horstmann (2001a) as *Aritranis fuscicornis* (Tschek, 1871)
	*Cryptus luctuosus holalpinus* Heinrich, 1951	A	Schwarz (2015)
	*Enclisis ruficeps* (Desvignes, 1856)	N	New record (M. Schwarz det.)
	*Enclisis schwarzi* Bordera & Hernández-Rodríguez, 2003	N	New record (M. Schwarz det.)
	*Gambrus aphrodite* (Heinrich, 1949)	H	In Horstmann (2001a) as *Hoplocryptus aphrodite* Heinrich, 1949
	*Gambrus bipunctatus* (Tschek, 1872)	A	Schmidt and Zmudzinski (2003)
	*Gambrus ornatus* (Gravenhorst, 1829)	A	Schwarz (2005)
	*Hidryta simplex* (Tschek, 1871)	A	Schwarz (2005)
	*Hoplocryptus besseianus* (Seyrig, 1926)	A	Schwarz (2007)
	*Hoplocryptus bohemani* (Holmgren, 1856)	H	In Horstmann (2001a) as *Hoplocryptus rufoniger* (Desvignes, 1856)
	*Hoplocryptus centricolor* (Aubert, 1964)	A	Schwarz (2007)
	*Hoplocryptus heliophilus* (Tschek, 1871)	A	Schwarz (2007)
	*Hoplocryptus melanocephalus* (Gravenhorst, 1829)	A	Schwarz (2007)
	*Ischnus migrator* (Fabricius, 1775)	A	Horstmann (2001b)
	*Listrognathus firmator* (Fabricius, 1798)	H	In Horstmann (2001a) as *Listrognathus ligator* (Gravenhorst, 1829)
	*Meringopus fuscescens* (Gmelin, 1790)	H	In Horstmann (2001a) as *M. cyanator* (Gravenhorst, 1829)
	*Meringopus turanus* (Habermehl, 1918)	A	Schwarz (2020)
	*Pycnocryptodes insinuator* (Gravenhorst, 1829)	H	In Horstmann (2001a) as *Pycnocryptodes freygessneri* (Schmiedeknecht, 1904)
	*Schreineria populnea* (Giraud, 1872)	N	New record (M. Riedel det.)
	*Thrybius brevispina* (Thomson, 1896)	N	New record (M. Schwarz det.)
	*Trychosis ambigua* (Tschek, 1871)	H	In Horstmann (2001a) as *Trychosis mesocastana* (Tschek, 1871)
** Ctenopelmatinae **			
	*Ctenopelma nigripenne* (Gravenhorst, 1829)	A	Riedel et al. (2013)
	*Ctenopelma tomentosum* (Desvignes, 1856)	H	In Horstmann (2001a) as *Ctenopelma athimi* Kriechbaumer, 1896
	*Homaspis alpigena* (Strobl, 1903)	A	Riedel (2018a)
	*Homaspis analis* (Holmgren, 1857)	A	Aubert (2000)
	*Notopygus flavicornis* Holmgren, 1857	H	In Horstmann (2001a) as *Homaspis flavicornis* (Holmgren, 1857)
	*Notopygus minkii* Vollenhoven, 1878	H	In Horstmann (2001a) as *Notopygus bicarinatus* Teunissen, 1953
	*Xenoschesis ustulata* (Desvignes, 1856)	H	In Horstmann (2001a) as *Xenoschesis resplendens* (Holmgren, 1857)
	*Gunomeria macrodactylus* (Holmgren, 1856)	A	Horstmann (2008a)
	*Hadrodactylus flavofacialis* Horstmann, 2000	A	Horstmann (2008a)
	*Hypsantyx lituratoria* (Linnaeus, 1761)	H	In Horstmann (2001a) as *Hypsantyx lituratorius* (Linnaeus, 1761)
	*Phobetes atricoxator* Aubert, 2007	A	Aubert (2007)
	*Syndipnus alutaceus* (Holmgren, 1857)	A	Aubert (2000)
	*Syndipnus lilianeator* Aubert, 2007	A	Aubert (2007)
	*Syndipnus polyblastoides* (Kriechbaumer, 1897)	A	Horstmann (2001c)
	*Syndipnus sternoleucus* (Gravenhorst, 1829)	A	Aubert (2000)
	*Synodites decipiens* (Woldstedt, 1877)	H	In Horstmann (2001a) as *Syndipnus decipiens* (Woldstedt, 1877)
	*Alexeter hypargyrici* (Hinz, 1996)	H	In Horstmann (2001a) as *Mesoleius hypargyrici* Hinz, 1996
	*Alexeter segmentarius* (Fabricius, 1787)	A	Schmidt and Zmudzinski (2003)
	*Barytarbes flavicornis* (Thomson, 1892)	H	In Horstmann (2001a) as *Barytarbes segmentarius* (Perkins, 1962)
	*Barytarbes pectoralis* (Brischke, 1871)	A	Riedel et al. (2013)
	*Barytarbes superbus* Schmiedeknecht, 1914	A	Schmidt and Zmudzinski (2003)
	*Campodorus autumnalis* (Woldstedt, 1874)	H	In Horstmann (2001a) as *Mesoleius autumnalis* Woldstedt, 1874
	*Campodorus boreator* Kasparyan, 2006	A	Riedel (2018a)
	*Campodorus ciliator* Kasparyan, 2006	A	Riedel (2018a)
	*Campodorus deletus* (Thomson, 1894)	A	Riedel (2018a)
	*Campodorus elegans* (Parfitt, 1882)	H	In Horstmann (2001a) as *Campodorus pineti* (Thomson, 1893)
	*Campodorus formosus* (Gravenhorst, 1829)	A	Horstmann (2008a)
	*Campodorus fuscotrochanteratus* (Strobl, 1903)	H	In Horstmann (2001a) as *Mesoleius fuscotrochanteratus* Strobl, 1903
	*Campodorus mediosanguineus* (Heinrich, 1950)	H	In Horstmann (2001a) as *Mesoleius mediosanguineus* Heinrich, 1950
	*Campodorus nigriventris* Kasparyan, 2005	A	Kasparyan (2005)
	*Campodorus pectinator* Kasparyan, 2003	A	Kasparyan (2005)
	*Himerta bisannulata* (Thomson, 1883)	H	In Horstmann (2001a) as *Himerta pfeifferi* (Bauer, 1939)
	*Lagarotis alpina* (Strobl, 1903)	H	In Horstmann (2001a) as *Lagarotis erythrocerops* Heinrich, 1949
	*Lagarotis pubescens* (Holmgren, 1857)	H	In Horstmann (2001a) as *Alexeter pubescens* (Holmgren, 1857)
	*Mesoleius assiduus* Holmgren, 1876	H	In Horstmann (2001a) as *Campodorus*assiduus (Holmgren, 1876)
	*Mesoleius axillaris* (Stephens, 1835)	H	In Horstmann (2001a) as *Campodorus axillaris* (Stephens, 1835)
	*Mesoleius efferus* Holmgren, 1876	H	In Horstmann (2001a) as *Campodorus efferus* (Holmgren, 1876)
	*Mesoleius euphrosyne* Teunissen, 1953	A	Riedel (2018a)
	*Mesoleius fuscipes* Holmgren, 1857	H	In Horstmann (2001a) as *Campodorus fuscipes* (Holmgren, 1857)
	*Mesoleius intermedius* (Gravenhorst, 1829)	H	In Horstmann (2001a) as *Campodorus intermedius* (Gravenhorst, 1829)
	*Mesoleius lindemansi* Teunissen, 1953	A	Kasparyan (2000)
	*Mesoleius peronatus* (Marshall, 1876)	H	In Horstmann (2001a) as *Campodorus crassitarsis* (Thomson, 1883)
	*Mesoleius strobli* Habermehl, 1925	A	Schmidt and Zmudzinski (2003)
	*Mesoleius urbanus* Teunissen, 1945	H	In Horstmann (2001a) as *Mesoleius ribesii* Bauer, 1961
	*Neostroblia ruficollis* (Holmgren, 1857)	H	In Horstmann (2001a) as *Mesoleius ruficollis* Holmgren, 1857
	*Otlophorus exareolator* Aubert, 2007	A	Aubert (2007)
	*Otlophorus nervellator* Aubert, 2007	A	Aubert (2007)
	*Otlophorus nigricoxator* Aubert, 2007	A	Aubert (2007)
	*Otlophorus rufogibbosus* (Kriechbaumer, 1897)	H	In Horstmann (2001a) as *Alexeter rufogibbosus* (Kriechbaumer, 1897)
	*Perispuda bignellii* (Bridgman, 1881)	H	In Horstmann (2001a) as *Perispuda flavitarsis* (Thomson, 1893)
	*Rhinotorus jussilai* Reshchikov, 2016	A	Riedel (2018a)
	*Saotis compressiuscula* (Thomson, 1883)	A	Kasparyan and Kopelke (2009)
	*Saotis granulator* Kasparyan & Kopelke, 2010	A	Kasparyan and Kopelke (2009)
	*Saotis morleyi* Fitton, 1976	A	Kasparyan and Kopelke (2009)
	*Saotis pygidiator* Kasparyan & Kopelke, 2009	A	Kasparyan and Kopelke (2009)
	*Saotis renovata* (Morley, 1911)	A	Kasparyan and Kopelke (2009)
	*Saotis tricolor* (Thomson, 1883)	H	In Horstmann (2001a) as *Saotis liopleuris* (Thomson, 1888)
	*Scopesis flavopicta* (Gravenhorst, 1829)	H	In Horstmann (2001a) as *Mesoleius flavopictus* (Gravenhorst, 1829)
	*Scopesis tarsatae* Horstmann, 2006	A	Horstmann (2006b)
	*Bremiella pulchella* (Kriechbaumer, 1890)	N	New record (A. Humala det.)
	*Lathiponus semiluctuosus* (Vollenhoven, 1878)	H	In Horstmann (2001a) as *Lathiponus bicolor* (Brischke, 1878)
	*Lathrolestes buccinator* (Holmgren, 1857)	H	In Horstmann (2001a) as *Perilissus buccinator* Holmgren, 1857
	*Lathrolestes erythrocephalus* (Gravenhorst, 1829)	H	In Horstmann (2001a) as *Perilissus erythrocephalus* (Gravenhorst, 1829)
	*Lathrolestes tripunctor* (Thunberg, 1824)	H	In Horstmann (2001a) as *Perilissus tripunctor* (Thunberg, 1824)
	*Perilissus banaticus* (Kiss, 1924)	H	In Horstmann (2001a) as *Perilissus amperis* (Heinrich, 1949)
	*Perilissus holmgreni* Habermehl, 1925	A	Schmidt and Zmudzinski (2003)
	*Perilissus punctatissimus* Strobl, 1903	A	Horstmann (2001c)
	*Zaplethocornia exstinctor* Aubert, 1985	A	Aubert (2000)
	*Asthenara scabricula* (Thomson, 1894)	A	Riedel (2018a)
	*Glyptorhaestus pumilus* Hinz, 1975	A	Schmidt and Zmudzinski (2003)
	*Lethades lapponicus* (Holmgren, 1857)	A	Horstmann (2008a)
	*Lethades punctatissimus* (Strobl, 1903)	A	Horstmann (2001c)
	*Pion nigripes* Schiødte, 1839	H	In Horstmann (2001a) as *Pion crassipes* (Holmgren, 1857)
	*Rhorus amauronemati* Kasparyan, 2017	A	Kasparyan (2017)
	*Rhorus angulatus* (Thomson, 1888)	A	Aubert (2000)
	*Rhorus austriator* Aubert, 1988	A	Schmidt and Zmudzinski (2003)
	*Rhorus blennocampae* Kasparyan, 2017	A	Kasparyan (2017)
	*Rhorus brunnifemur* Kasparyan, 2015	A	Riedel (2018a)
	*Rhorus carinifer* Kasparyan, 2019	A	Kasparyan (2019)
	*Rhorus dineurae* Kasparyan, 2017	A	Kasparyan (2017)
	*Rhorus emarginatus* Kasparyan, 2019	A	Kasparyan (2019)
	*Rhorus neuter* Aubert, 1988	A	Kasparyan (2017)
	*Rhorus pristiphorae* Kasparyan, 2014	A	Kasparyan (2017)
	*Rhorus subfasciatus* (Stephens, 1835)	A	Kasparyan (2019)
	*Rhorus trochanteratus* Kasparyan, 2015	A	Kasparyan (2017)
	*Rhorus xanthopygus* Kasparyan, 2014	A	Riedel (2018a)
	*Seleucus cuneiformis* Holmgren, 1860	A	Vikberg and Koponen (2000)
** Cylloceriinae **			
	*Allomacrus longecaudatus* (Strobl, 1903)	N	New record (A. Humala det.)
	*Cylloceria sylvestris* (Gravenhorst, 1829)	A	Schmidt et al. (2012)
	*Rossemia longithorax* Humala, 1997	N	New record (A. Humala det.)
** Diplazontinae **			
	*Campocraspedon annulitarsis* (Hedwig, 1938)	A	Klopfstein (2014)
	*Diplazon angustus* Dasch, 1964	A	Schmidt and Zmudzinski (2009)
	*Diplazon pallicoxa* Manukyan, 1987	A	Klopfstein (2014)
	*Fossatyloides gracilentus* (Holmgren, 1858)	H	In Horstmann (2001a) as *Syrphoctonus gracilentus* (Holmgren, 1858)
	*Homotropus crassicornis* Thomson, 1890	H	In Horstmann (2001a) as *Syrphoctonus crassicornis* (Thomson, 1890)
	*Homotropus dimidiatus* (Schrank, 1802)	H	In Horstmann (2001) as *Syrphoctonus dimidiatus* (Schrank, 1802)
	*Homotropus elegans* (Gravenhorst, 1829)	H	In Horstmann (2001a) as *Syrphoctonus elegans* (Gravenhorst, 1829)
	*Homotropus frontorius* (Thunberg, 1824)	H	In Horstmann (2001a) as *Syrphoctonus fissorius* (Gravenhorst, 1829)
	*Homotropus haemorrhoidalis* Szépligeti, 1898	H	In Horstmann (2001a) as *Syrphoctonus haemorrhoidalis* (Szépligeti, 1898)
	*Homotropus incisus* Thomson, 1890	H	In Horstmann (2001a) as *Syrphoctonus incisus* (Thomson, 1890)
	*Homotropus longiventris* Thomson, 1890	H	In Horstmann (2001a) as *Syrphoctonus longiventris* (Thomson, 1890)
	*Homotropus megaspis* Thomson, 1890	H	In Horstmann (2001a) as *Syrphoctonus megaspis* (Thomson, 1890)
	*Homotropus nigritarsus* (Gravenhorst, 1829)	H	In Horstmann (2001a) as *Syrphoctonus nigritarsus* (Gravenhorst, 1829)
	*Homotropus pallipes* (Gravenhorst, 1829)	H	In Horstmann (2001a) as *Syrphoctonus pallipes* (Gravenhorst, 1829)
	*Homotropus pictus* (Gravenhorst, 1829)	H	In Horstmann (2001a) as *Syrphoctonus pictus* (Gravenhorst, 1829)
	*Homotropus signatus* (Gravenhorst, 1829)	H	In Horstmann (2001a) as *Syrphoctonus signatus* (Gravenhorst, 1829)
	*Homotropus strigator* (Fabricius, 1793)	H	In Horstmann (2001a) as *Syrphoctonus strigator* (Fabricius, 1793)
	*Homotropus sundevalli* (Holmgren, 1858)	H	In Horstmann (2001a) as *Syrphoctonus sundevalli* (Holmgren, 1858)
	*Sussaba aciculata* (Ruthe, 1859)	A	Riedel (2018a)
	*Sussaba placita* Dasch, 1964	A	Klopfstein (2014)
	*Sussaba roberti* Klopfstein, 2014	A	Klopfstein (2014)
	*Syrphophilus asperatus* Dasch, 1964	A	Riedel (2018a)
	*Tymmophorus erythrozonus* (Förster, 1850)	H	In Horstmann (2001a) as *Tymmophorus rufiventris* (Gravenhorst, 1829)
	*Tymmophorus suspiciosus* (Brischke, 1871)	A	Schmidt and Zmudzinski (2009)
	*Woldstedtius merkli* Vas, 2016	A	Müller (2020a), det. S. Klopfstein
** Hybrizontinae **			
	*Hybrizon ghilarovi* Tobias, 1988	A	Riedel (2018a)
	*Ogkosoma cremieri* (Romand, 1838)	H	In Horstmann (2001a) as *Eurypterna cremieri* (Roman, 1838)
** Ichneumoninae **			
	*Alomya debellator* (Fabricius, 1775)	H	Transferred from Alomyinae to Ichneumoninae, Alomyini by Bennett et al. (2019)
	*Alomya punctalata* (Schellenberg, 1802)	H	Transferred from Alomyinae to Ichneumoninae, Alomyini by Bennett et al. (2019)
	*Alomya pygmaea* Heinrich, 1949	H	Transferred from Alomyinae to Ichneumoninae, Alomyini by Bennett et al. (2019)
	*Alomya semiflava* Stephens, 1835	H	Transferred from Alomyinae to Ichneumoninae, Alomyini by Bennett et al. (2019)
	*Anisobas platystylus* Thomson, 1888	A	Horstmann (2007)
	*Anisobas rebellis* Wesmael, 1845	H	In Horstmann (2001a) as *Anisobas jugorum* Heinrich, 1949
	*Aethecerus foveolatus* Gregor, 1940	A	Riedel (2018a)
	*Aethecerus subuliferus* (Holmgren, 1890)	H	In Horstmann (2001a) as *Phaeogenes subuliferus* Holmgren, 1890
	*Aoplus mustela* (Kriechbaumer, 1895)	A	Bauer (2001)
	*Apaeleticus vibicariae* (Kriechbaumer, 1888)	H	In Horstmann (2001a) as *Platylabus vibicariae* Kriechbaumer, 1888
	*Barichneumon nubilis* (Brischke, 1891)	N	New record (M. Riedel det.)
	*Barichneumon scopulatus* Tereshkin, 2004	N	New record (M. Riedel det.)
	*Coelichneumon biguttorius* (Thunberg, 1789)	H	In Horstmann (2001a) as *Coelichneumon comitator* (Linnaeus, 1758)
	*Coelichneumon eburnifrons* (Wesmael, 1857)	H	In Horstmann (2001a) as *Coelichneumon pumilionobilis* Heinrich, 1951
	*Coelichneumon erythromerus* (Rudow, 1888)	A	Horstmann (2000a)
	*Coelichneumon funebrator* Horstmann, 2006	A	Horstmann (2006c)
	*Coelichneumon ophiusae* (Kriechbaumer, 1890)	H	In Horstmann (2001a) as *Coelichneumon bodmanorum* Heinrich, 1950
	*Coelichneumon probator* Horstmann, 2000	A	Horstmann (2000a)
	*Coelichneumon quadriannulatus* (Gravenhorst, 1829)	A	Riedel (2012)
	*Coelichneumon torsor* (Thunberg, 1824)	A	Riedel (2012)
	*Cratichneumon flavifrons* (Schrank, 1781)	A	Schmidt and Zmudzinski (2005)
	*Cratichneumon lancea* (Dalla Torre, 1901)	H	In Horstmann (2001a) as Barichneumon lancea (Dalla Torre, 1901)
	*Cratichneumon sexarmillatus* (Kriechbaumer, 1891)	H	In Horstmann (2001a) as *Cratichneumon albiscuta* (Thomson, 1893)
	*Crypteffigies tenuicinctus* (Schmiedeknecht, 1928)	A	Schmidt and Zmudzinski (2003)
	*Ctenichneumon phragmitecolator* Bauer, 2001	A	Bauer (2001)
	*Cyclolabus axillatorius* (Thunberg, 1824)	H	In Horstmann (2001a) as *Hoplismenus axillatorius* (Thunberg, 1824)
	*Dicaelotus montanus* (de Stefani, 1885)	H	Schmidt and Zmudzinski (2005)
	*Dicaelotus orbitalis* Thomson, 1891	A	Schmidt and Zmudzinski (2003)
	*Dicaelotus pudibundus* (Wesmael, 1845)	A	Riedel et al. (2013)
	*Dicaelotus punctiventris* (Thomson, 1891)	A	Bauer (2001)
	*Dicaelotus pusillator* (Gravenhorst, 1807)	A	Schmidt and Zmudzinski (2003)
	*Dicaelotus schmiedeknechti* Diller & Shaw, 2014	A	Diller and Shaw (2014)
	*Dirophanes coryphaeus* (Wesmael, 1845)	H	In Horstmann (2001a) as *Phaeogenes coryphaeus* Wesmael, 1845
	*Dirophanes foveolatus* (Perkins, 1953)	H	In Horstmann (2001a) as *Phaeogenes foveolatus* Perkins, 1953
	*Dirophanes mysticus* (Wesmael, 1855)	H	In Horstmann (2001a) as *Phaeogenes mysticus* Wesmael, 1855
	*Dirophanes regenerator* (Fabricius, 1804)	H	In Horstmann (2001a) as *Dirophanes rusticatus* (Wesmael, 1845)
	*Diphyus longigena* (Thomson, 1888)	A	Schmidt and Zmudzinski (2003)
	*Diphyus politus* (Wesmael, 1855)	N	New record (M. Riedel det.)
	*Diphyus restitutor* (Wesmael, 1859)	N	New record (M. Riedel det.)
	*Epitomus alpicola* (Strobl, 1901)	H	In Horstmann (2001a) as *Epitomas alpicola* (Strobl, 1901)
	*Epitomus proximus* Perkins, 1953	A	Horstmann and Floren (2001)
	*Heterischnus bicolorator* (Aubert, 1965)	A	Schmidt and Zmudzinski (2003)
	*Heterischnus coxator* (Thomson, 1891)	A	Schmidt and Zmudzinski (2003)
	*Heterischnus filiformis* (Gravenhorst, 1829)	A	Schmidt and Zmudzinski (2005)
	*Homotherus coxistriatus* Riedel, 2020	A	Riedel (2020)
	*Ichneumon deliratorius* Linnaeus, 1758	H	In Horstmann (2001a) as *Coelichneumon deliratorius* (Linnaeus, 1758)
	*Ichneumon lugens* Gravenhorst, 1829	H	In Horstmann (2001a) as *Chasmias lugens* (Gravenhorst, 1829)
	*Ichneumon oblongus* Schrank, 1802	A	Horstmann (2001b)
	*Mevesia alternans* (Wesmael, 1845)	H	In Horstmann (2001a) as *Phaeogenes alternans* Wesmael, 1845
	*Neischnus germari* (Ratzeburg, 1849)	H	In Horstmann (2001a) as *Neischnus oxypygus* Heinrich, 1952
	*Orgichneumon calcatorius* (Thunberg, 1824)	A	Schmidt and Zmudzinski (2003)
	*Phaeogenes bacilliger* Kriechbaumer, 1891	A	Horstmann and Floren (2008)
	*Phaeogenes nigridens* Wesmael, 1845	H	In Horstmann (2001a) as *Tycherus nigridens* (Wesmael, 1845)
	*Phaeogenes pfefferi* Teunissen, 1972	A	Schmidt and Zmudzinski (2003)
	*Platylabus baueri* Riedel, 2008	A	Riedel (2008)
	*Platylabus borealis* Holmgren, 1871	A	Schmidt and Zmudzinski (2003)
	*Platylabus curtorius* (Thunberg, 1824)	H	In Horstmann (2001a) as *Platylabus eurygaster* Holmgren, 1871
	*Platylabus daemon* Wesmael, 1845	H	In Horstmann (2001a) as *Asthenolabus daemon* (Wesmael, 1845)
	*Platylabus daemonops* (Heinrich, 1944	A	Riedel (2008)
	*Platylabus heteromallus* (Berthoumieu, 1910)	A	Riedel (2008)
	*Platylabus judaicus* Berthoumieu, 1900	A	Riedel (2008)
	*Platylabus mesoleucus* (Heinrich, 1936)	H	In Horstmann (2001a) as *Asthenolabus mesoleucus* (Heinrich, 1936)
	*Platylabus minor* Riedel, 2008	A	Riedel (2008)
	*Platylabus neglectus* (Fonscolombe, 1847)	H	In Horstmann (2001a) as *Platylabus decipiens* Wesmael, 1848
	*Platylabus obator* (Desvignes, 1856)	A	Riedel (2008)
	*Platylabus oehlkei* Heinrich, 1972	A	Riedel (2008)
	*Platylabus orbitalis* (Gravenhorst, 1829)	H	In Horstmann (2001a) as *Platylabus muticus* Thomson, 1894, *P. suborbitalis* Kriechbaumer, 1894 and *P. volubilis* (Gravenhorst, 1829)
	*Platylabus perexiguus* Heinrich, 1973	A	Riedel (2018a)
	*Platylabus pseudomuticus* Riedel, 2008	A	Riedel (2008)
	*Platylabus pseudopumilio* Riedel, 2008	A	Riedel (2008)
	*Platylabus ruficoxatus* Riedel, 2008	A	Riedel (2008)
	*Platylabus stalii* Holmgren, 1871	H	In Horstmann (2001a) as *Asthenolabus stalii* (Holmgren, 1871)
	*Platylabus sternoleucus* Wesmael, 1853	H	In Horstmann (2001a) as *Asthenolabus sternoleucus* (Wesmael, 1853)
	*Pristicerops laetepictus* (Costa, 1863)	A	Schmidt and Zmudzinski (2005)
	*Probolus crassulus* Horstmann, 2000	A	Horstmann (2000b)
	*Rhadinodonta rufidens* (Wesmael, 1845)	N	New record (M. Riedel det.)
	*Rubicundiella externa* (Berthoumieu, 1895)	A	Schmidt and Zmudzinski (2003)
	*Rubicundiella walli* Riedel, 2020	A	Riedel (2020)
	*Spilothyrateles nuptatorius* (Fabricius, 1793)	H	In Horstmann (2001a) as *Spilothyrateles fabricii* (Schrank, 1802)
	*Stenobarichneumon basalis* (Perkins, 1960)	A	Schmidt and Zmudzinski (2003)
	*Syspasis carinator* (Fabricius, 1798)	H	In Horstmann (2001a) as *Syspasis helleri* (Holmgren, 1878)
	*Syspasis puerulus* (Kriechbaumer, 1890)	N	New record (M. Riedel det.)
	*Trachyarus anceps* (Berthoumieu, 1906)	A	Gokhman (2007)
	*Trachyarus brevipennis* Roman, 1918	A	Gokhman (2007))
	*Trachyarus decipiens* Gokhman, 2007	A	Gokhman (2007)
	*Trachyarus solyanikovi* Gokhman, 2007	A	Gokhman (2007)
	*Tycherus amaenus* (Wesmael, 1845)	H	In Horstmann (2001a) as *Phaeogenes amaenus* Wesmael, 1845
	*Tycherus brunneus* (Kiss, 1924)	A	Riedel (2018a)
	*Tycherus capitosus* (Holmgren, 1890)	A	New record (E. Diller det.)
	*Tycherus dodecellae* Ranin, 1983	A	New record (E. Diller det.)
	*Tycherus horstmanni* Diller, 2006	A	Diller (2006)
	*Tycherus impiger* (Wesmael, 1845)	H	In Horstmann (2001a) as *Phaeogenes impiger* Wesmael, 1845
	*Tycherus jucundus* (Wesmael, 1845)	H	In Horstmann (2001a) as *Phaeogenes jucundus* Wesmael, 1845
	*Tycherus longicarinus* Diller, 2006	A	Diller (2006)
	*Tycherus maxi* Diller, 2009	A	Diller and Zwakhals (2009)
	*Tycherus nigrifemoratus* Ranin, 1983	A	Riedel (2018a)
	*Tycherus parvitor* Aubert, 1982	A	Riedel (2018a)
	*Tycherus planipectus* (Holmgren, 1890)	H	In Horstmann (2001a) as *Phaeogenes planipectus* Holmgren, 1890
	*Tycherus septentrionalis* (Holmgren, 1890)	A	New record (E. Diller det.)
	*Tycherus socialis* (Ratzeburg, 1852)	H	In Horstmann (2001a) as *Tycherus discoidalis* (Ratzeburg, 1852)
	*Tycherus stockerorum* Diller, 2008	A	Diller et al. (2008)
	*Tycherus teres* (Berthoumieu, 1899)	A	Horstmann and Floren (2001)
	*Virgichneumon perversus* (Kriechbaumer, 1893)	H	In Horstmann (2001a) as *Barichneumon perversus* (Kriechbaumer, 1893)
** Mesochorinae **			
	*Astiphromma aggressor* (Fabricius, 1804)	H	In Horstmann (2001a) as *Astiphromma marginellum* (Holmgren, 1860)
	*Astiphromma alpinum* (Roman, 1909)	H	In Horstmann (2001a) as *Astiphromma dispersum* Schwenke, 1999
	*Astiphromma flagellator* Riedel, 2015	A	Riedel (2015)
	*Astiphromma hirsutum* (Bridgman, 1883)	H	In Horstmann (2001a) as *Astiphromma granigerum* (Thomson, 1886)
	*Astiphromma italicum* Schwenke, 1999	A	Riedel (2015)
	*Astiphromma striatum* (Brischke, 1880)	H	In Horstmann (2001a) as *Astiphromma mandibulare* (Thomson, 1886)
	*Astiphromma tridentatum* Schwenke, 1999	A	Riedel (2015)
	*Cidaphus areolatus* (Boie, 1850)	H	In Horstmann (2001a) as *Cidaphus brischkei* (Szépligeti, 1911)
	*Dolichochorus longiceps* (Strobl, 1904)	H	In Horstmann (2001a) as *Astiphromma longiceps* (Strobl, 1904) (Broad & Watanabe 2019)
	*Mesochorus alpigenus* Strobl, 1904	H	In Horstmann (2001a) as *Mesochorus compactus* Schwenke, 1999
	*Mesochorus anhalthinus* Schwenke, 2002	H	In Horstmann (2001a) as *Mesochorus obscurus* Schwenke, 1999
	*Mesochorus arenarius* (Haliday, 1838)	H	In Horstmann (2001a) as *Mesochorus nigripes* Ratzeburg, 1852
	*Mesochorus arthridion* Araujo & Vivallo, 2015	H	Syn.: *Mesochorus artus* Schwenke, 1999 (Araujo & Vivallo, 2015)
	*Mesochorus atriventris* Cresson, 1872	A	Horstmann (2006a)
	*Mesochorus britannicus* Schwenke, 1999	A	Riedel (2018b)
	*Mesochorus cimbicis* Ratzeburg, 1844	H	In Horstmann (2001a) as *Mesochorus confusus* Holmgren, 1860
	*Mesochorus crassimanus* Holmgren, 1860	A	Horstmann (2006a)
	*Mesochorus cyparissiae* Schwenke, 2002	H	In Horstmann (2001a) as *Mesochorus parilis* Schwenke, 1999
	*Mesochorus errabundus* Hartig, 1838	H	Schmidt et al. (2012)
	*Mesochorus faciator* Horstmann, 2003	A	Horstmann (2003b)
	*Mesochorus fluvialis* Schwenke, 2002	A	Schwenke (2002)
	*Mesochorus fulgurator* Horstmann, 2006	A	Horstmann (2006a)
	*Mesochorus fulvoides* Riedel, 2018	A	Riedel (2018b)
	*Mesochorus giberius* (Thunberg, 1824)	H	Syn.: *Mesochorus temporalis* Thomson, 1886 (Riedel and Kolarov in press)
	*Mesochorus heterodon* Horstmann, 2006	A	Horstmann (2006a)
	*Mesochorus iugosus* Schwenke, 2002	A	Schwenke (2002)
	*Mesochorus iwatensis* (Uchida, 1928)	A	Riedel (2018a)
	*Mesochorus jenensis* Schwenke, 2002	A	Schwenke (2002)
	*Mesochorus jugicola* Strobl, 1904	H	In Horstmann (2001a) as *Mesochorus caliginosus* Schwenke, 1999
	*Mesochorus laricis* Hartig, 1838	A	Horstmann (2006a)
	*Mesochorus lilioceriphilus* Schwenke, 2000	A	Haye and Kenis (2004)
	*Mesochorus marginatus* Thomson, 1886	N	New record (M. Riedel det.)
	*Mesochorus nemus* Schwenke, 2002	A	Schwenke (2002)
	*Mesochorus olerum* Curtis, 1833	H	In Horstmann (2001a) as *Mesochorus pectoralis* Ratzeburg, 1844
	*Mesochorus pelvis* Schwenke, 2002	A	Schwenke (2002)
	*Mesochorus perticatus* Schwenke, 1999	A	Riedel (2007)
	*Mesochorus perugianus* Schwenke, 1999	H	In Horstmann (2001a) as *Mesochorus plebejanus* Schwenke, 1999 (Riedel and Kolarov in press)
	*Mesochorus plumosus* Dasch, 1971	A	Riedel (2018b)
	*Mesochorus provocator* Aubert, 1965	A	Riedel (2018b)
	*Mesochorus sulphuripes* Brischke, 1880	A	Schmidt et al. (2012)
	*Mesochorus thomsonii* Dalla Torre, 1901	A	(Riedel and Kolarov in press)
	*Mesochorus tibialis* Schwenke, 2002	A	Schwenke (2002)
	*Mesochorus tipularius* Gravenhorst, 1829	H	In Horstmann (2001a) as *Mesochorus minutus* Szépligeti, 1914
	*Mesochorus trifoveatus* Schwenke, 2004	A	Riedel (2018b)
	*Mesochorus veluminis* Schwenke, 1999	A	Schwenke (2002)
	*Mesochorus vittator* (Zetterstedt, 1838)	N	New record (M. Riedel det.)
** Metopiinae **			
	*Exochus argutus* Tolkanitz, 1993	A	Riedel (2018a)
	*Exochus fletcheri* Bridgman, 1884	A	Schmidt et al. (2010)
	*Exochus hirsutus* Tolkanitz, 1993	A	Riedel (2018a)
	*Hypsicera ecarinata* Tolkanitz, 1986	N	New record (A. Humala det.)
	*Metopius citratus* (Geoffroy, 1785)	H	In Horstmann (2001a) as *Metopius dissectorius* (Panzer, 1805)
	*Metopius necatorius* (Fabricius, 1793)	H	In Horstmann (2001a) as *Metopius connexorius* Wesmael, 1849
	*Scolomus borealis* (Townes, 1971)	A	Schmidt et al. (2010)
	*Stethoncus sulcator* Aubert, 1965	A	Broad and Shaw (2005)
	*Triclistus facialis* Thomson, 1887	A	Riedel (2018a)
** Neorhacodinae **			
	*Neorhacodes enslini* (Ruschka, 1922)	H	Recognised as separate subfamily by Broad et al. (2018)
** Ophioninae **			
	*Enicospilus intermedius* Johannson, 2018	N	New record (J. Müller det.)
	*Enicospilus myricae* Broad & Shaw, 2016	A	Kopetz et al. (2019)
	*Ophion angularis* Johansson in Johansson & Cederberg, 2019	N	New record (N. Johansson det.)
	*Ophion artemisiae* Boie, 1855	A	Johansson and Cederberg (2019)
	*Ophion borealis* Johansson in Johansson & Cederberg, 2019	A	Theunert (2020)
	*Ophion brevicornis* Morley, 1915	A	Schmidt et al. (2012)
	*Ophion brocki* Johansson in Johansson & Cederberg, 2019	A	Johansson and Cederberg (2019)
	*Ophion confusus* Johansson in Johansson & Cederberg, 2019	A	Johansson and Cederberg (2019)
	*Ophion costatus* Ratzeburg, 1848	A	Schmidt et al. (2012)
	*Ophion ellenae* Johansson in Johansson & Cederberg, 2019	N	New record (N. Johansson det.)
	*Ophion inclinans* Johansson in Johansson & Cederberg, 2019	N	New record (N. Johansson det.)
	*Ophion perkinsi* Brock, 1982	A	Schmidt et al. (2012)
	*Ophion sylvestris* Johansson in Johansson & Cederberg, 2019	N	New record (N. Johansson det.)
	*Ophion tenuicornis* Johansson in Johansson & Cederberg, 2019	A	Johansson and Cederberg (2019)
	*Ophion variegatus* Rudow, 1883	N	New record (N. Johansson det.)
** Orthocentrinae **			
	*Aniseres subarcticus* Humala, 2007	N	New record (A. Humala det.)
	*Batakomacrus flaviceps* (Gravenhorst, 1829)	A	Dalla Torre (1902), Kirchner (1867), confirmed by A.Humala
	*Batakomacrus subarcticus* Humala, 2010	N	New record (A. Humala det.)
	*Batakomacrus sylvicola* Humala, 2010	N	New record (A. Humala det.)
	*Eusterinx minima* (Strobl, 1903)	A	Humala (2004))
	*Eusterinx refractaria* Rossem, 1982	A	Humala (2004)
	*Helictes fabularis* Rossem, 1987	N	New record (A. Humala det.)
	*Megastylus similis* Dasch, 1992	N	New record (A. Humala det.)
	*Megastylus suecicus* Rossem, 1983	A	Riedel (2007)
	*Neurateles compressus* (Thomson, 1897)	A	Schmidt et al. (2012)
	*Orthocentrus castellanus* Aubert, 1981	N	New record (A. Humala det.)
	*Orthocentrus hirsutor* Aubert, 1969	A	Schmidt et al. (2012)
	*Orthocentrus strigatus* Holmgren, 1858	N	New record (A. Humala det.)
	*Orthocentrus winnertzii* Förster, 1850	A	Humala et al. (2020)
	*Plectiscidea aquilonia* Humala, 2008	N	New record (A. Humala det.)
	*Plectiscidea zonata* (Gravenhorst, 1829)	H	In Horstmann (2001a) as *Proclitus zonatus* (Gravenhorst, 1829)
	*Stenomacrus cognatus* (Holmgren, 1858)	A	Horstmann (2008a)
	*Stenomacrus deletus* (Thomson, 1897)	A	Schmidt et al. (2012)
	*Stenomacrus exserens* (Thomson, 1898)	A	Horstmann (2008a)
	*Stenomacrus holmgreni* (Kirchner, 1867)	H	In Horstmann (2001a) as *Stenomacrus lapponicus* Horstmann & Yu, 1999
	*Stenomacrus inferior* Aubert, 1981	N	New record (A. Humala det.)
	*Stenomacrus palustris* (Holmgren, 1858)	H	In Horstmann (2001a) as *Stenomacrus paluster* (Holmgren, 1858)
	*Stenomacrus ungula* (Thomson, 1897)	N	New record (A. Humala det.)
	*Symplecis apicola* Förster, 1871	A	Horstmann (1988) (missing in Horstmann (2001a), but unknown if intentional or erroneously)
** Orthopelmatinae **			
	*Orthopelma brevicorne* Morley, 1907	N	New record (A. Humala det.)
** Phygadeuontinae **			
	*Atractodes ficticius* (Förster, 1876)	H	In Horstmann (2001a) as *Atractodes genuinus* (Förster, 1876)
	*Atractodes helveticus* (Förster, 1876)	H	In Horstmann (2001a) as *Atractodes oreophilus* Förster, 1876
	*Atractodes incrassator* Roman, 1926	A	Jussila (2001)
	*Atractodes punctator* Roman, 1909	A	Jussila (2001)
	*Atractodes remotus* Jussila, 1979	A	Jussila (2001)
	*Cephalobaris eskelundi* Kryger, 1915	A	Horstmann (2012c)
	*Ceratophygadeuon italicus* Horstmann, 1979	A	Riedel (2018a)
	*Ceratophygadeuon parvicaudator* (Aubert, 1965)	A	Schwarz and Shaw (2010)
	*Ceratophygadeuon varicornis* (Thomson, 1885)	H	In Horstmann (2001a) as *Ceratophygadeuon maritimus* Horstmann, 1979
	*Charitopes leucobasis* Townes, 1983	A	Schmidt and Zmudzinski (2003)
	*Clypeoteles distans* (Thomson, 1884)	A	Riedel (2018a)
	*Cremnodes montanus* (Förster, 1876)	H	In Horstmann (2001a) as *Stilpnus montanus* (Förster, 1876)
	*Eudelus gumperdensis* (Schmiedeknecht, 1897)	A	Horstmann (2008a)
	*Eudelus mediovittata* (Schmiedeknecht, 1897)	H	In Horstmann (2001a) as *Acrolyta mediovittata* (see Schwarz & Shaw 2000)
	*Eudelus pallicarpus* (Thomson, 1884)	N	New record (M. Schwarz det.)
	*Eudelus scabriculus* (Thomson, 1884)	A	Schmidt and Zmudzinski (2003)
	*Gelis albopilosus* Schwarz, 2002	A	Schwarz (2002)
	*Gelis alopecosae* Horstmann, 1986	N	New record (M. Schwarz det.)
	*Gelis cayennator* (Thunberg, 1824)	H	In Horstmann (2001a) as *Gelis brassicae* Horstmann, 1986
	*Gelis fuscicornis* (Retzius, 1783)	H	In Horstmann (2001a) as *Gelis longulus* (Zetterstedt, 1838)
	*Gelis imitatus* Schwarz, 2016	A	Schwarz (2016)
	*Gelis mitis* Schwarz, 1994	A	Riedel (2018a)
	*Gelis orbiculatus* (Gravenhorst, 1829)	A	In Horstmann (2001a) as synonym of *G. areator* (Panzer, 1804) (Schwarz 2016)
	*Gelis ornatus* (Thomson, 1884)	A	New record (M. Schwarz det.)
	*Gelis parens* Schwarz, 1998	A	Riedel (2007)
	*Gelis recens* Schwarz, 2002	A	Schwarz (2002)
	*Gelis rubricollis* (Thomson, 1884)	A	Schwarz (2016)
	*Gelis rufibasalis* Schwarz, 2016	A	Schwarz (2016)
	*Gelis sapporoensis* (Ashmead, 1906)	A	Schwarz (2016)
	*Gelis shawidaani* Schwarz, 2002	A	Schwarz (2002)
	*Gelis spinula* (Thomson, 1884)	A	Schwarz (2002)
	*Gelis terribilis* Schwarz, 2002	A	Schwarz (2002)
	*Gelis vicinus* (Gravenhorst, 1829)	H	In Horstmann (2001a) as *Blapsidotes vicinus* (Gravenhorst, 1829)
	*Gelis zeirapherator* (Aubert, 1966)	N	New record (M. Schwarz det.)
	*Grasseiteles punctus* (Holmgren, 1857)	H	In Horstmann (2001a) as *Diaglyptellana punctata* Horstmann, 1986
	*Hemiteles maricesca* Schwarz & Shaw, 2000	A	Schwarz and Shaw (2000)
	*Hemiteles rubropleuralis* Kiss, 1929	N	New record (M. Schwarz det.)
	*Holcomastrus bituberculatus* (Schmiedeknecht, 1905)	H	In Horstmann (2001a) as *Fianoniella bituberculata* (Schmiedeknecht, 1905)
	*Isadelphus longisetosus* (Schmiedeknecht, 1897)	A	Horstmann and Floren (2001)
	*Isadelphus minutus* Horstmann, 2009	A	Horstmann (2009b)
	*Isadelphus tuberculatus* Horstmann, 2009	N	New record (M. Schwarz det.)
	*Mastrus longicauda* Horstmann, 1990	A	Horstmann and Floren (2001)
	*Mesoleptus congener* (Förster, 1876)	A	Jussila et al. (2010), Riedel et al. (2013)
	*Mesoleptus devotus* (Förster, 1876)	A	Jussila et al. (2010), Riedel et al. (2013)
	*Mesoleptus incessor* (Haliday, 1838)	H	In Horstmann (2001a) as *Mesoleptus annexus* (Förster, 1876)
	*Mesoleptus laticinctus* (Walker, 1874)	H	In Horstmann (2001a) as *Mesoleptus filicornis* (Thomson, 1884)
	*Mesoleptus solitarius* (Förster, 1876)	A	Jussila et al. (2010)
	*Mesoleptus tobiasi* Jonaitis, 2004	A	Riedel (2007)
	*Odontoneura quercicola* Horstmann, 2012	A	Horstmann (2012c)
	*Orthizema gracilicornutum* Schwarz, 2018	A	Schwarz (2018)
	*Orthizema obscurum* Horstmann, 1993	A	Riedel (2018a)
	*Phygadeuon atricolor* Horstmann, 2001	A	Horstmann (2001d)
	*Phygadeuon atropos* Kriechbaumer, 1892	A	Horstmann (2001d)
	*Phygadeuon austriacus* (Gravenhorst, 1829)	A	Horstmann (2001d)
	*Phygadeuon bavaricus* Horstmann, 2001	A	Horstmann (2001d)
	*Phygadeuon brevitarsis* Thomson, 1884	A	Horstmann (2001d)
	*Phygadeuon depressus* Horstmann, 2012	A	Horstmann (2012c)
	*Phygadeuon dromicus* (Gravenhorst, 1815)	A	Sebald and Goßner (2006)
	*Phygadeuon exannulatus* (Strobl, 1904)	A	Schmidt and Zmudzinski (2003)
	*Phygadeuon fraternae* Horstmann, 2001	A	Horstmann (2001d)
	*Phygadeuon habermehli* Roman, 1930	A	Horstmann (2001d)
	*Phygadeuon laevipleuris* Horstmann, 2001	A	Horstmann (2001d)
	*Phygadeuon leucostigmus* Gravenhorst, 1829	A	Schmidt and Zmudzinski (2003)
	*Phygadeuon macrocephalus* Horstmann, 2001	A	Horstmann (2001d)
	*Phygadeuon melanopygus* (Gravenhorst, 1829)	H	In Horstmann (2001a) as *Theroscopus melanopygus* (Gravenhorst, 1829)
	*Phygadeuon neoflavicans* Horstmann, 1967	A	Horstmann (2008a)
	*Phygadeuon nigrifemur* Horstmann, 2001	A	Horstmann (2001d)
	*Phygadeuon praealpinus* Horstmann, 2001	A	Horstmann (2001d)
	*Phygadeuon unidentatus* Horstmann, 2001	A	Horstmann (2001d)
	*Pygocryptus brevicornis* (Brischke, 1881)	H	In Horstmann (2001a) as *Pygocryptus grandis* (Thomson, 1884)
	*Stibeuon infernalis* (Ruthe, 1859)	H	In Horstmann (2001a) as *Stibeutes infernalis* (Ruthe, 1859)
	*Stibeutes breviareolatus* (Thomson, 1884)	A	Horstmann (2010)
	*Stibeutes brevicornis* (Lange, 1911)	A	Horstmann (2010)
	*Stibeutes buccatus* Horstmann, 2010	A	Horstmann (2010)
	*Stibeutes coriaceus* Horstmann, 2010	A	Horstmann (2010)
	*Stibeutes heterogaster* (Thomson, 1885)	H	In Horstmann (2001a) as *Stibeutes gravenhorstii* Förster, 1850
	*Stibeutes rozsypali* (Gregor, 1941)	A	Horstmann (2010)
	*Sulcarius fuscicarpus* (Thomson, 1885)	A	Riedel et al. (2013)
	*Thaumatogelis femoralis* (Brischke, 1881)	A	Schwarz (2001)
	*Thaumatogelis inexspectatus* Schwarz, 2001	A	Schwarz (2001)
	*Thaumatogelis innoxius* Schwarz, 2001	A	Schwarz (2001)
	*Theroscopus fasciatulus* Horstmann, 1979	A	New record (M. Schwarz det.)
	*Theroscopus megacentrus* (Schiødte, 1839)	H	In Horstmann (2001a) as *Theroscopus ornaticornis* (Schmiedeknecht, 1897)
	*Theroscopus opacinotum* (Hellén, 1967)	A	Horstmann (2000c)
	*Theroscopus praeacutus* Schwarz, 2018	A	Schwarz (2018)
	*Theroscopus pullator* (Gravenhorst, 1829)	H	In Horstmann (2001a) as *Orthizema pullator* (Gravenhorst, 1829)
	*Theroscopus ripa* Schwarz, 2018	A	Schwarz (2018)
	*Tropistes burakowskii* (Sawoniewicz, 1996)	A	Riedel (2018a)
	*Zoophthorus infirmus* (Gravenhorst, 1829)	H	In Horstmann (2001a) as *Eudelus infirmus* (Gravenhorst, 1829)
	*Zoophthorus trochanteralis* (Dalla Torre, 1902)	H	In Horstmann (2001a) as *Theroscopus trochanteralis* (Dalla Torre, 1901)
** Pimplinae **			
	*Acrodactyla carinator* (Aubert, 1965)	A	Bauer (2002)
	*Acrodactyla similis* Horstmann, 2011	A	Horstmann (2011a)
	*Clistopyga canadensis* Provancher, 1880	H	Schmidt and Zmudzinski (2003)
	*Dolichomitus excavatus* Zwakhals, 2010	A	Zwakhals (2010)
	*Dolichomitus milleri* Zwakhals, 2010	A	Zwakhals (2010)
	*Dolichomitus quercicolus* Zwakhals, 2010	A	Zwakhals (2010)
	*Dolichomitus scutellaris* (Thomson, 1877)	A	Zwakhals (2010)
	*Ephialtes zirnitsi* Ozols, 1962	A	Bauer (2002), Horstmann (2008b)
	*Flavopimpla cicatricosa* (Ratzeburg, 1848)	H	In Horstmann (2001a) as *Afrephialtes cicatricosus* (Ratzeburg, 1848)
	*Iania pictifrons* (Thomson, 1877)	H	In Horstmann (2001a) as *Dreisbachia pictifrons* (Thomson, 1877)
	*Megaetaira madida* (Haliday, 1838)	H	In Horstmann (2001a) as *Acrodactyla madida* (Haliday, 1839)
	*Pimpla flavicoxis* Thomson, 1877	A	Several German records of *Pimpla aquilonia* auct. nec Cresson, 1870 belong to this species
	*Pimpla insignatoria* (Gravenhorst, 1807)	H	In Horstmann (2001a) as *Pimpla coxalis* Habermehl, 1917
	*Pimpla nigrohirsuta* Strobl, 1902	A	Horstmann (2001c)
	*Polysphincta vexator* Fitton, Shaw & Gauld, 1988	A	Müller (2020b)
	*Pseudorhyssa alpestris* (Holmgren, 1860)	H	Formerly placed in Pseudorhyssini (Poemeniinae, Klopfstein et al. 2019)
	*Pseudorhyssa nigricornis* (Ratzeburg, 1852)	H	Formerly placed in Pseudorhyssini (Poemeninae, Klopfstein et al. 2019)
	*Reclinervellus nielseni* (Roman, 1923)	A	Horstmann (2003a)
	*Scambus euphrantae* (Schmiedeknecht, 1914)	A	Riedel et al. (2013)
	*Scambus inanis* (Schrank, 1802)	H	In Horstmann (2001a) as *Scambus annulatus* (Kiss, 1924)
	*Theronia atalantae* (Poda, 1761)	H	Formerly placed in Pimplini, now in Theroniini (Klopfstein et al. 2019)
	*Theronia laevigata* (Tschek, 1869)	H	Formerly placed in Pimplini, now in Theroniini (Klopfstein et al. 2019)
	*Tromatobia lineatoria* (Villers, 1789)	A	Horstmann (2008a)
	*Zabrachypus primus* Cushman, 1920	A	Müller (2020b)
	*Zatypota anomala* (Holmgren, 1860)	H	In Horstmann (2001a) as *Sinarachna anomala* (Holmgren, 1860)
** Poemeniinae **			
	*Neoxorides striatus* Johansson, 2020	A	Johansson (2020)
	*Neoxorides varipes* (Holmgren, 1860)	A	Riedel (2018a)
** Rhyssinae **			
	*Rhyssa kriechbaumeri* Ozols, 1973	A	Horstmann (2002b)
** Tersilochinae **			
	*Allophroides platyurus* (Strobl, 1904)	A	Riedel et al. (2013)
	*Astrenis brunneofacies* Vikberg, 2000	A	Schmidt et al. (2010)
	*Astrenis nigrifacies* Vikberg, 2000	A	Schmidt et al. (2010)
	*Gelanes carinatus* Khalaim & Blank, 2011	A	Khalaim and Blank (2011)
	*Gelanes clavulatus* Khalaim & Blank, 2011	A	Khalaim and Blank (2011)
	*Gelanes simillimus* Horstmann, 1981	A	Khalaim and Blank (2011)
	*Phradis polonicus* Horstmann, 1981	A	Khalaim et al. (2009)
	*Phrudus defectus* Stelfox, 1966	A	Khalaim et al. (2009)
	*Probles caudiculatus* Khalaim, 2007	A	Khalaim (2007)
	*Tersilochus cognatus* (Holmgren, 1860)	A	Horstmann (2008a)
** Tryphoninae **			
	*Cladeutes discedens* (Woldstedt, 1874)	A	Schmidt and Zmudzinski (2003)
	*Netelia longipes* (Brauns, 1889)	A	Schmidt and Zmudzinski (2003)
	*Phytodietus bayeri* (Gregor, 1935)	A	Riedel (2018a)
	*Phytodietus continuus* Thomson, 1877	A	Riedel et al. (2013)
	*Phytodietus elongator* Aubert, 1963	A	Riedel (2018a)
	*Phytodietus femoralis* Holmgren, 1860	A	Riedel et al. (2013)
	*Phytodietus rhodopaeus* Kolarov, 2003	N	New record (A. Humala det.)
	*Cteniscus devius* (Mason, 1955)	A	Horstmann (2008a)
	*Ctenochira angulata* (Thomson, 1883)	A	Schmidt and Zmudzinski (2003)
	*Ctenochira breviseta* (Ratzeburg, 1852)	H	In Horstmann (2001a) as *Ctenochira aberrans* (Ruthe, 1855)
	*Ctenochira grossa* (Brischke, 1871)	N	New record (M. Riedel det.)
	*Ctenochira magnusi* Haraldseide, 2018	A	Haraldseide (2018)
	*Ctenochira meridionator* Aubert, 1969	A	Riedel (2018a)
	*Ctenochira sphaerocephala* (Gravenhorst, 1829)	H	In Horstmann (2001a) as *Ctenochira sphaerocephalus* (Gravenhorst, 1829)
	*Dyspetes arrogator* Heinrich, 1949	A	Schmidt and Zmudzinski (2003)
	*Dyspetes luteomarginatus* Habermehl, 1925	H	In Horstmann (2001a) as *Dyspetes praerogator* (Thomson, 1883)
	*Erromenus nitens* Strobl, 1903	H	In Horstmann (2001a) as *Erromenus tarsator* Aubert, 1969
	*Grypocentrus apicalis* Thomson, 1883	A	Schönrogge and Altenhofer (1992)
	*Polyblastus nanus* Kasparyan, 1973	A	Riedel (2002)
	*Polyblastus stenocentrus* Holmgren, 1857	A	Riedel et al. (2013)
	*Polyblastus tuberculatus* Teunissen, 1953	A	Riedel (2002)
	*Smicroplectrus bucculatus* Kasparyan, 1990	A	Kasparyan and Khalaim (2007)
	*Tryphon alpinator* Horstmann, 2012	A	In Heinrich (1949) as *Tryphon alpinus* Strobl, 1903 (Horstmann 2012b)
	*Tryphon latrator* (Fabricius, 1781)	H	In Horstmann (2001a) as *Tryphon auricularis* Thomson, 1883
	*Tryphon zavreli* Gregor, 1939	A	Schmidt and Zmudzinski (2003)
** Xoridinae **			
	*Xorides propinquus* (Tschek, 1869)	A	Riedel (2018a)
	*Xorides rusticus* (Desvignes, 1856)	H	In Horstmann (2001a) as *Xorides bischoffi* (Clément, 1938)
